# Radiation dose-rate effects on gene expression for human biodosimetry

**DOI:** 10.1186/s12920-015-0097-x

**Published:** 2015-05-12

**Authors:** Shanaz A. Ghandhi, Lubomir B. Smilenov, Carl D. Elliston, Mashkura Chowdhury, Sally A. Amundson

**Affiliations:** Center for Radiological Research, Columbia University, VC11-237, 630 West 168th Street, New York, NY 10032 USA

**Keywords:** Low dose-rate effects, Radiation biodosimetry, Prediction

## Abstract

**Background:**

The effects of dose-rate and its implications on radiation biodosimetry methods are not well studied in the context of large-scale radiological scenarios. There are significant health risks to individuals exposed to an acute dose, but a realistic scenario would include exposure to both high and low dose-rates, from both external and internal radioactivity. It is important therefore, to understand the biological response to prolonged exposure; and further, discover biomarkers that can be used to estimate damage from low-dose rate exposures and propose appropriate clinical treatment.

**Methods:**

We irradiated human whole blood *ex vivo* to three doses, 0.56 Gy, 2.23 Gy and 4.45 Gy, using two dose rates: acute, 1.03 Gy/min and a low dose-rate, 3.1 mGy/min. After 24 h, we isolated RNA from blood cells and these were hybridized to Agilent Whole Human genome microarrays. We validated the microarray results using qRT-PCR.

**Results:**

Microarray results showed that there were 454 significantly differentially expressed genes after prolonged exposure to all doses. After acute exposure, 598 genes were differentially expressed in response to all doses. Gene ontology terms enriched in both sets of genes were related to immune processes and B-cell mediated immunity. Genes responding to acute exposure were also enriched in functions related to natural killer cell activation and cell-to-cell signaling. As expected, the p53 pathway was found to be significantly enriched at all doses and by both dose-rates of radiation. A support vectors machine classifier was able to distinguish between dose-rates with 100 % accuracy using leave-one-out cross-validation.

**Conclusions:**

In this study we found that low dose-rate exposure can result in distinctive gene expression patterns compared with acute exposures. We were able to successfully distinguish low dose-rate exposed samples from acute dose exposed samples at 24 h, using a gene expression-based classifier. These genes are candidates for further testing as markers to classify exposure based on dose-rate.

**Electronic supplementary material:**

The online version of this article (doi:10.1186/s12920-015-0097-x) contains supplementary material, which is available to authorized users.

## Background

To optimize biodosimetry methods for estimating radiation exposure after a large-scale radiological event, all likely radiation qualities, modes of exposure and exposure times should be considered while designing assays that will be useful for triage [1]. It is important to determine whether an individual received a dose by a lower dose-rate, which can be from both internal and external sources of radiation, and which may pose a moderate health risk as compared with a single acute dose. Low dose-rates of exposure may also confound the estimation of total dose if dosimetry assays are not tailored to distinguish dose-rate effects [2].

There have been many studies addressing the development of a gene expression-based signature for estimation of dose, in peripheral blood irradiated *ex vivo* [3–6], in blood from total body irradiated (TBI) patients [7–9], isolated human monocytes [10], CD4+ lymphocytes [11], skin from biopsies [12, 13], and cell lines from humans [14–16]; and a few that address effects of similar doses delivered over a period of hours or days in cell lines [15, 16], but little is known about the gene expression response of human blood to low dose-rates (LDR). Development of a gene signature in blood that is able to discriminate between irradiated samples without a matching pre-exposure sample has been shown to be a powerful tool in biodosimetry assay development [3], and the goal of this study was to use a similar approach and identify genes that would discriminate between both dose and dose-rates. There are *in vivo* studies on transcriptomic changes in radiation workers; and also changes induced by internal emitters in mice, that have determined dose and dose-rate effects in organs and blood [17–23]. These studies have revealed that gene expression differences can be detected after prolonged exposure times.

In the study presented in this paper, exposure of human blood *ex vivo* to LDR and acute irradiation gave a robust gene expression response as measured by microarrays and validated by qRT-PCR. We identified genes that responded uniquely to LDR and not to acute doses. Class prediction by dose-rate successfully identified samples as LDR-exposed or acute. This is an important first step towards developing and further refining gene-expression based assays that can be used to determine the contribution of dose-rate to overall dose.

## Methods

### Irradiation and culture of blood

We collected blood from healthy volunteers (5 females and 3 males) between the ages of 26 and 59 years, with informed consent in compliance with the Columbia University Institutional Review Board (protocol approval number IRB-AAAF2671). 27 mL of blood from each donor was collected into Sodium Citrate tubes (Becton Dickinson, New Jersey, catalog# 366415) and mixed well. Blood was diluted in equal volumes of RPMI solution (supplemented with 10 % heat-inactivated fetal bovine serum and 1 % penicillin streptomycin) prior to irradiations in 50 mL Tube Spin® Bioreactor 50 tubes (TPP, Switzerland), which are optimized for culture incubation and gas exchange.

All irradiations were performed in an X-Rad 320 Biological Irradiator (Precision X-Ray, North Branford CT). This device provides a system for precise delivery of radiation doses to specimens in a self-contained, shielded cabinet, and features an adjustable shelf, exchangeable beam hardening filters, and a programmable control panel that allows tube current ranging from 0.1 to 12.5 mA at its maximum. To achieve the lowest possible dose-rate using this device, we designed and built a custom Thoraeus filter (1.25 mm Sn, 0.25 mm Cu, 1.5 mm Al). This filter provides a dose rate of ~4 Gy/day at the maximum SSD (source-to-surface distance), and a dose rate of ~1Gy/min, an acceptable “acute” dose rate, at 40 cm SSD. This custom beam filter was designed to enable both acute and low dose rate irradiations to be performed using the same quality of x rays, while changing only the mA and SSD.

**Fig. 1:**
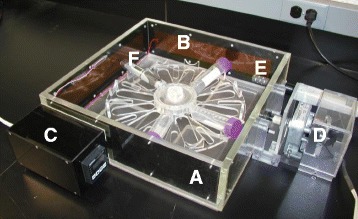
Custom incubator for low-dose *ex vivo* blood irradiations, consisting of (**a**) Incubator chamber containing the rotating sample platform; (**b**) Heating elements attached to the chamber walls; (**c**). Temperature controller; (**d**) Turntable motor; (**e**) CO_2_ and humidity monitor; and (**f**) Temperature and humidity logger. The sample platform completes three rotations per hour to provide dose homogeneity. The lid that seals this chamber during use is removed in this image

The protracted irradiations of blood samples also required the maintenance of a tissue culture environment, with control of temperature, humidity, and carbon dioxide content. Commercially available incubators were deemed unsuitable, due to the large amounts of metal in their construction. We did not want any metal in the x-ray beam because the increased scatter would affect dose homogeneity. We therefore, created an all plastic incubator (Fig. 1) capable of incubating blood in 50 ml conical tubes, angled to maintain a higher surface area to volume ratio for efficient gas exchange, and to keep the blood within a 20 cm diameter target area in order to minimize planar dose variation. The samples were rotated at a speed of three rotations per hour to further minimize any dose inhomogeneity. Temperature was controlled through solid state heaters on a feedback loop attached to carbon fiber walls to distribute the heat evenly. This setting maintained a temperature of 37 °C (±0.5 °C) for 24 h. The CO_2_ concentration and humidity were maintained by perfusing the incubator at a rate of 1.7 l/min with a gas mix (5 % CO_2_ and 95 % air) that was humidified using a bubble humidifier and monitored directly by numeric readouts from a GMP70 Hand-held CO_2_ meter (Vaisala, Finland). The temperature was also monitored directly by the readout of the solid-state heater controller. Temperature and relative humidity were also recorded using a data-logger (EL-USB-2-LCD, Lascar Electronics, Inc., Erie, PA). For irradiations, the incubator was placed in the X-ray machine and the incubation parameters were allowed to stabilize before blood samples (in 50 ml tubes) were placed in the sample holders.

Acute exposures were performed at a dose rate of 1.03 Gy/min x rays at a machine setting of 320 keV/12.5 mA. The samples were exposed to doses of 0.56 Gy, 2.23 Gy and 4.45 Gy and then returned to the cell culture incubator at 37 °C, 5 % CO_2_ for the rest of the 24 h incubation. For low dose-rate exposures, blood samples were exposed to 0.56 Gy (3 h at 3.1 mGy/min, followed by 21 h in a standard incubator), 2.23 Gy (12 h at 3.1 mGy/min, followed by 12 h in a standard incubator), and 4.45 Gy (24 h at 3.1 mGy/min). RNA was isolated from all samples at 24 h after the start of exposure. All irradiated samples were compared with a matching sham-irradiated control sample from the same donor.

### Microarray analyses

RNA was isolated 24 h after the start of exposure following the recommended protocol for the PerfectPure RNA kit from 5Prime (Gaithersburg, MD). Globin transcripts were reduced using the Ambion GLOBINclear-Human kit (Life Technologies, Grand Island, NY, catalog# AM1980). RNA yields were quantified using the NanoDrop ND1000 Spectrophotometer (Thermo Scientific) and RNA quality was checked using the 2100 Bioanalyzer (Agilent Technologies). RNA used for microarray hybridization had an RNA Integrity Number of >8.5.

Cyanine-3 (Cy3) labeled cRNA was prepared with the One-Color Low Input Quick Amp Labeling Kit (Agilent Technologies) according to the manufacturer’s instructions. Dye incorporation and cRNA yield were verified with the NanoDrop ND1000 Spectrophotometer; 1.6 microgram of cRNA, >9 pmol Cy3 per microgram cRNA was fragmented and hybridized (17 h with rotation at 65 °C) to Agilent Whole Human Genome Microarrays 4X44K v2 (G4112F), and then washed using the Gene Expression Hybridization Kit and GE Wash Buffers as recommended by Agilent. Slides were then scanned with the Agilent DNA Microarray Scanner (G2505B), and the images were analyzed (Agilent Feature Extraction Software ver. 10.7) with default parameters for background correction and flagging non-uniform features.

Background-corrected hybridization intensities were imported into BRB-ArrayTools, Version 4.2.1 [24] log2-transformed and median normalized; after normalization the distribution of signals remained uniform across all arrays. Non-uniform outliers or features not significantly above background intensity in 25 % or more of the hybridizations were filtered out. A further filter requiring a minimum 1.4 fold change in at least 20 % of the hybridizations was then applied to remove genes with no variation across the dataset, with >10,000 genes remaining which is optimal for data analyses; probes were also averaged to one probe per gene and duplicate genes were reduced by selecting the one with maximum signal intensity, yielding a final set of 12,073 features that were used for subsequent analyses. The microarray data is available through the Gene Expression Omnibus with accession number GSE65292.

RNA samples from five donors (3 male and 2 female to mitigate against the possibility of sex-specific bias in results [25, 26] were hybridized. A total of 35 RNA samples were hybridized in this study. Class comparison analyses were conducted using BRB-ArrayTools to identify genes that were differentially expressed between classes using a random-variance *t*-test [27]. Genes with *p*-values less than 0.001 were considered statistically significant. The false discovery rate (FDR) was also estimated for each gene using the method of Benjamini and Hochberg [28], to control for false positives.

### Quantitative RT-PCR

The High-Capacity cDNA Archive Kit (Life Technologies, Foster City, CA) was used to prepare cDNA from total RNA from three of the female donors not used in the microarray hybridization experiments. Quantitative real-time RT-PCR (qRT-PCR) was performed for selected genes using Taqman assays (Life Technologies) on a Low Density array (384-well microfluidic card), to confirm microarray experiment findings for selected genes. The 48 genes and corresponding assays are listed in Additional file 1. In gene expression validation studies, 400 ng cDNA was used as input for PCR. Quantitative real time PCR reactions were performed with the ABI 7900 Real Time PCR System using Universal PCR Master Mix (Life Technologies), with initial activation at 50 °C for 120 s and 94.5 °C for 10 min, followed by 40 cycles of 97 °C for 30 s and 59.7 °C for 60 s. Relative fold-change was calculated by the ΔΔC_T_ method, using SDS version 2.3 (Thermofisher). Data were normalized to *RPLPO* gene expression levels. We used Genorm [29] to assess the stability of the housekeeping genes included on the Low Density array panels, and *RPLPO* gene expression was found to be most stable in our data. *RPLPO* was therefore used to normalize the qRT-PCR data.

### Gene Ontology and pathway analyses

Lists of genes significantly over- or under-expressed relative to controls were imported separately into the PANTHER database (version 9.0, release 2014-01-24) to identify enriched biological themes and gene ontology (GO) terms using the statistical overrepresentation test, GO-Slim Biological Processes, Molecular Functions and Pathways [30]. Benjamini corrected *p*- values <0.05 were considered significant.

### Class prediction analysis

Gene sets for class prediction were determined using BRB-ArrayTools Class Predictions, which provide various options for classifier prediction and cross-validation. Predictions used a cut off significance p-value of 0.0001 (for dose-rate and irradiation classification) and 0.001 (for dose classification) between classes to determine the classifier gene set. Support Vector Machines [31] was used for classification of samples between two categories, and Diagonal Linear Discriminant Analysis, which avoids complex models with excessive parameters in order to avoid over fitting data without loss of performance [32] was used for classifications with more than two categories. The algorithms tested the classifier gene set for accuracy and sensitivity and specificity [24] and we used the Leave-one-out cross-validation method to compute mis-classification rates.

## Results

### Microarray results

We analyzed gene expression changes using the BRB-ArrayTools Class comparison tool for paired analyses between classes and the results of significantly differentially expressed genes (*p* <0.001) are summarized in Table 1. We found the broadest changes and highest number of genes affected at 4.45 Gy by both dose-rates (354 genes changed after LDR 4.45 Gy exposure and 565 genes changed after acute 4.45 Gy exposures at 24 h); Additional file 2 contains details of gene expression changes summarized in Table 1. An intersection of the differentially expressed genes at each dose (Additional file 3) indicated that the set of differentially expressed genes after 4.45 Gy included most of the genes changed at lower doses.

**Table 1:** Summary of genes differentially changed (*p* <0.001) in various class comparisons

Class comparison	Number of genes significantly changed	False discovery rate	Number of up regulated genes	Number of down regulated genes
**LDR 4 Gy vs 0 Gy**	354	0.03	236	118
**Acute 4 Gy vs 0 Gy**	565	0.02	354	211
**LDR 2 Gy vs 0 Gy**	213	0.06	134	79
**Acute 2 Gy vs 0 Gy**	205	0.06	150	55
**LDR 0.5 Gy vs 0 Gy**	71	0.16	49	22
**Acute 0.5 Gy vs 0 Gy**	65	0.18	55	10
**LDR 4 Gy vs Acute 4 Gy**	243	0.10	116	127

We performed gene ontology analyses on the sets of differentially expressed genes. Among the sets of genes responding to 0.56 or 2.23 Gy at low dose rate, PANTHER GO-slim analysis reveal only one significantly affected function after LDR 0.56 Gy exposure, DNA repair (with a p-value of 3.7 X10^-2^). Genes in this category were *XPC*, *DDB2*, *POLH*, *GADD45A* and *PCNA*, all of which are sensitive radiation response genes.

The genes responding to the 4.45 Gy dose at both dose rates showed additional significantly enriched biological processes. A comparison of significantly enriched biological terms and the genes belonging to each category are shown in Table 2. Cellular processes, immune processes, B cell mediated immunity and cell communication were common biological functions affected by both low-dose rate and acute exposure. Unique to the low dose-rate response genes, was the pyrimidine nucleobase metabolic process (*p*-value 2.7 × 10^-2^) with genes involved in DNA editing. After acute 4.45 Gy exposures, biological processes affected included natural killer cell activation (*p*-value 6.7 × 10^-5^) and other cell signaling processes (*p*-value 1.3 × 10^-2^), not observed after LDR. GO-slim molecular functions revealed that receptor activity and binding were significantly affected by both LDR 4.45 Gy (*p*-value 2.2 × 10^-3^) and acute 4.45 Gy (*p*-value 1.8 × 10^-11^) doses, with an additional molecular function category of chemokines (*p*-value 1.3 × 10^-2^) significant only after acute exposure, not observed after LDR.

**Table 2:** Biological process enrichment analysis using PANTHER

PANTHER GO-Slim Biological Process	LDR 4 Gy	LDR 4 Gy	Acute 4 Gy	Acute 4 Gy
	genes	*p*-value	genes	*p*-value
**Cellular process**	110	2.65E-04	199	5.98E-13
**Cell communication**	68	1.11E-03	112	1.45E-06
**Immune system process**	43	2.24E-03	78	9.19E-09
**Pyrimidine nucleobase metabolic process**	6	2.73E-02		NS^a^
**B cell mediated immunity**	11	4.10E-02	21	6.01E-06
**Response to stimulus**	38	4.66E-02	72	3.77E-07
**Immune response**		NS^a^	41	3.08E-08
**Natural killer cell activation**		NS^a^	13	6.69E-05
**Developmental process**		NS^a^	93	6.11E-04
**Cell-cell signaling**		NS^a^	35	1.31E-02

^a^ *NS* not significant

### Comparison of low-dose rate and acute exposure

We also directly compared the gene expression response to 24-hour continuous LDR exposure with that of the acute exposure at 4.45 Gy (Table 1). There were 243 genes differentially expressed when comparing the two different exposure rates, with a moderate range of fold change between 0.3 and 3.9. Gene ontology analysis of these 243 genes revealed enrichment of two processes: glycolysis (*p*-value 3.74 × 10^-4^) and monosaccharide metabolic process (*p*-value 2.5 × 10^-3^). Genes included in these two categories were members of the glycolysis pathway (lactate dehyrogenase A, *LDHA*; glyceraldehyde 3-phosphate dehydrogenase, *GAPDH*; 6-phospho-fructokinase type C, *PKFP*; enolase 1, *ENO1*; and hexokinase 2, *HK2*), all of which were expressed at lower levels in cells exposed to the protracted dose.

### Validation of gene expression by quantitative PCR

**Fig. 2:**
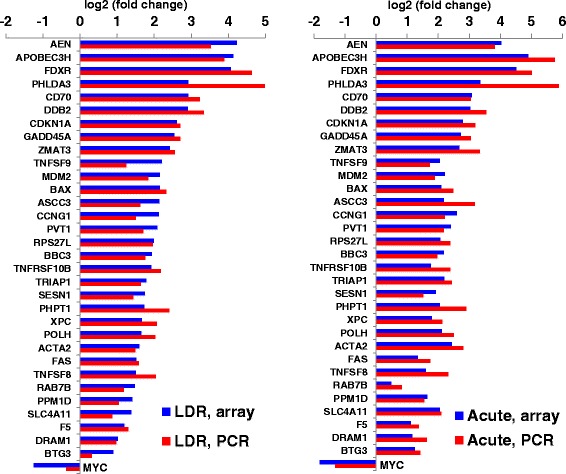
Validation of microarray results by qRT-PCR. Shown here are log_2_ (fold changes) of genes that were determined to be differentially regulated by the 4.45 Gy dose by both dose-rates. The graph on the left shows the mean log_2_ (fold change) after LDR 4.45 Gy; and the graph on the right is the mean of log_2_ (fold change) after Acute 4.45 Gy exposure. All microarray (blue bars, five biological replicates) and qRT-PCR (red-bars, three biological replicates) results are average fold-change from paired analyses; SEM values for all data are included in Additional file 4

We validated gene expression changes from microarrays using real time qRT-PCR in independent biological replicates. These samples were true independent biological replicates representing different donors from those used in the microarray hybridizations. We chose genes that were in common between all doses and dose-rates and are also known radiation response genes [3]. Fold changes by qRT-PCR agreed well with our microarray measurements and data are shown for both 4.45 Gy exposed groups (Fig. 2). We also validated gene expression levels for the lower doses (2.23 Gy and 0.56 Gy) and these data along with the 4.45 Gy results, mean and SEM, are included in Additional file 4.

### Gene expression patterns

**Fig. 3:**
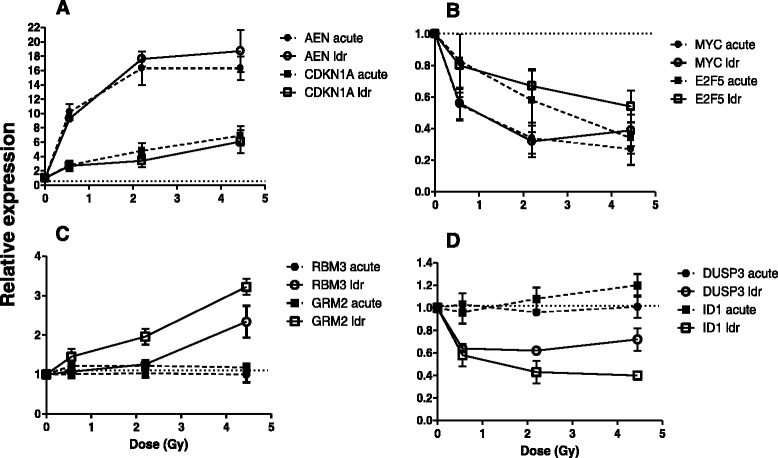
Patterns of gene expression response, shown as microarray results of representative genes, in all panels (**a**-**d**), open symbols with solid lines represent Low dose-rate (ldr) responses and closed symbols with dotted lines represent acute dose rate (acute) gene expression responses. **a** *AEN* (circles) and *CDKN1A* (squares) are representative genes in the group that showed similar up regulation of mRNA levels by both dose-rates. **b** *MYC* (circles) and *E2F5* (squares) are representative genes in the group that showed similar down regulation of mRNA levels by both dose-rates. **c** *RBM3* (circles) and *GRM2* (squares) are representative genes for the group that showed up regulation only by LDR. **d** *DUSP3* (circles) and *ID1* (squares) are representative genes for the group that showed down regulation only by LDR. All points are mean of 5 biological replicates (from paired analyses) and error bars are SEM

We searched for gene expression patterns that were in common between LDR and acute dose-rates and also those that showed differences that could distinguish samples that received a dose by a lower dose-rate. We identified more than 20 genes that showed a similar pattern of response where the dose rate appeared not to affect the changes. There were both up regulated and down regulated genes that belonged to this group and genes showing this characteristic behavior, *AEN* and *CDKN1A* (up in LDR and acute) and *MYC* and *E2F5* (down in both LDR and acute), are shown in Fig. 3a and b, respectively. The other pattern of interest was genes that only appeared to respond to LDR and not to acute dose. There were two types of genes in this group, one in which the genes were up regulated by LDR only, not acute doses (*RBM3* and *GRM2*, Fig. 3c) and the other in which genes were down regulated by LDR only, not acute doses (*DUSP3* and *ID2*, Fig. 3d). This preliminary assessment of different gene expression response patterns by LDR suggested that there are genes that could distinguish between the dose-rate for the same dose delivered. For some genes, such as *APOBEC3H*, *FDXR* and *PHLDA3*, induced at all doses by LDR and acute, the change in gene expression after the 4.45 Gy dose was higher in the acute dose group suggesting protection of response by low dose-rate.

### Class predictions

We used the Class Prediction tool in BRB-ArrayTools to determine a classifier that would correctly discriminate between dose-rates. Initially, we identified a classifier that distinguished between un-irradiated and acute or LDR-irradiated samples; in which a set of 121 genes (*p*-value <0.0001) correctly identified samples as 0 Gy control (CTL), Acute, or LDR with 94 % accuracy (Table 3) using Diagonal Linear Discriminant Analysis (DLDA). Next we tested and found that a 62-gene classifier could correctly classify exposed samples as Acute or LDR with 100 % accuracy using Support Vector Machines (Table 4). Lastly, we determined a classifier gene set comprised of 140 genes that correctly classified samples by dose without regard to the rate of exposure, with 90 % accuracy using Diagonal Linear Discriminant Analysis (Table 5). Details of classifier gene sets and performance of classifiers in cross-validations are included in Additional file 5; all classification analyses were performed on normalized gene expression signal intensities.

**Table 3:** Performance of the Diagonal Linear Discriminant Analysis classifier (121 genes) on irradiation and dose rate

Class	Sensitivity	Specificity
**Acute**	0.867	1
**0 Gy**	1	1
**LDR**	1	0.9

**Table 4:** Performance of the SVM machine classifier (62 genes) on dose rate

Class	Sensitivity	Specificity
**Acute**	1	1
**LDR**	1	1

**Table 5:** Performance of the Diagonal Linear Discriminant Analysis classifier (140 genes) on dose

Class	Sensitivity	Specificity
**0 Gy**	1	0.933
**0.56 Gy**	1	1
**2.23 Gy**	0.8	0.933
**4.45 Gy**	0.8	1

## Discussion

A variety of exposure types and combinations of exposures could result from an improvised nuclear device (IND) or radioactive dispersal device (RDD). In order to develop appropriate radiological triage approaches, biodosimetry assays must be tested and optimized for their ability to detect the contribution of various factors such as dose and dose-rate. A tiered approach to triage in large-scale scenarios [33, 34] would ideally include a low-dose rate detection assay to identify individuals who have received exposure over a prolonged time, so that their treatment can be adjusted accordingly. In a recent NATO study involving different laboratories that cross-validated results for different radiation biodosimetry assays, the dicentric chromosome assay, micronucleus assay, γ-H2AX and gene expression were assessed for their sensitivity and it was concluded that a combination of assays would be optimal for the estimation of dose and “never versus ever” exposure [35, 36]. Therefore, it is possible that a gene expression signature that can discriminate between low dose-rates and acute exposures, in combination with other assays that estimate dose, will enhance the ability to identify individuals with an immediate need for clinical treatment in a large-scale event.

Low dose-rate studies have also been done for very low cumulative doses, to assess the gene expression response. One such study on a prostate cancer cell line measured gene expression changes after a 24-hour chronic exposure to dose-rates as low as 7-17 μGy/min, and unexpectedly, found that the gene expression response was more similar to that of a 2 Gy acute dose than a 10 cGy acute dose [15]. In another study in mice, which were given a 5-week continuous dose of radiation at 2 μGy/min (cumulative dose 10.5 cGy, which was previously shown to be effective on gene expression as a single acute dose [6]) there was no significant change in gene expression. The effects observed at these very low doses may be the result of various factors inherent to the study design, the extended time in the second study or the origin and type of cells in the first, but it emphasizes the need for biodosimetry experiments to be designed to establish the range of dose rates and exposure times likely to impact on biodosimetric estimates and triage decisions.

Using an *ex vivo* blood irradiation model that has previously shown changes in radiation response genes that are sensitive to dose and time [3, 37], we exposed blood samples to relatively high total doses (up to 4 Gy), in the range of Acute Radiation Syndrome (ARS), either acutely, or over a period of 24 h. The prolonged radiation exposure time was to approximate exposure to fallout from an improvised nuclear device (IND) or radioactive dispersal device (RDD) that might occur before first responders arrive on the scene and are able to begin taking samples for biodosimetry.

In the current study, we detected gene expression changes at all doses, with increasing numbers of genes responding with increasing dose, as expected (Table 1). The number of genes differentially expressed at lower doses (0.56 Gy and 2.25 Gy) by LDR (*p*-value <0.001) was slightly higher than those after acute doses, however, the period of time from end of exposure to harvest was shorter after low dose-rate exposures, which may contribute to this difference. In the case of LDR 4.45 Gy however, the number of genes differentially expressed was less than acute 4.45 Gy (Additional file 2).

We focused our analyses on the 4.45 Gy dose responses, because they allowed us to determine and compare maximum differences in gene expression and biological function. These comparisons revealed that the acute exposure elicits many responses similar to LDR, but may also affect additional processes related to natural killer cells and cell-cell signaling via chemokines. In contrast, the gene expression response to LDR initiated some of the same functions as acute exposure, but additional processes related to nucleotide metabolism (Table 2) and DNA repair were also affected.

**Fig. 4:**
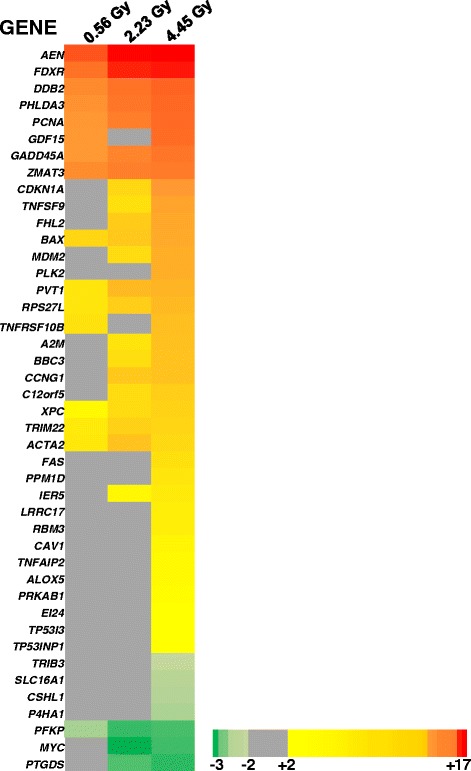
p53 pathway gene expression in LDR exposed blood cells. The genes contributing to the significance of the PANTHER p53-pathway are listed and their fold change depicted in the heat map, the three columns represent the mRNA changes at 24 h after 0.56 Gy, 2.23 Gy and 4.45 Gy dose; irradiation at low dose rate. Missing values that were not significant at a particular dose are shown in grey; the scale shows shades of yellow to red (upregulated genes) and shades of green (down-regulated genes)

Further GO analyses of the LDR gene expression response against PANTHER pathways revealed that the p53 pathway was found to be significantly affected at all doses (LDR 0.56Gy, p-value 5.0 × 10^-4^; LDR 2.23 Gy, p-value 4.8 × 10^-5^; and LDR 4.45 Gy, 1.5 × 10^-5^). This suggests that at 24 h after the start of exposure at all doses, p53-regulated functions in cell stress, cell cycle and DNA damage repair are important; even after the shortest LDR exposure. We focused on p53 target genes as this pathway was the top scoring biological pathway controlling gene regulation across all doses and dose-rates. The heat map in Fig. 4 depicts genes that are regulated by p53 across all three doses in the LDR treatment group. More p53-regulated genes were involved in the response after the highest dose (4.45 Gy) where blood cells were exposed continuously to the LDR radiation with no recovery period. In the case of the lowest dose (0.56 Gy) and intermediate dose (2.23 Gy) a subset of these genes responded and changes in mRNA were still detectable at 24 h after the start of exposure. This suggests that there is an accumulation of stress and damage during the protraction of dose, possibly mediated by p53 and also other transcriptional regulators [38, 39], that persists and is not completely resolved at 24 h after the start of exposure.

We were also able to identify groups of genes that were more responsive to LDR than acute dose rate (Fig. 3c and d). Genes such as *RBM3* and *GRM2* that were up regulated after LDR appeared to respond to doses ≥2.23 Gy. In the case of down regulated genes*, DUSP3* and *ID1*, the response was significant (*p*-value <0.001) at even the lowest dose, LDR 0.56 Gy. The down regulation of *ID1* mRNA after LDR was interesting because it is a known radiation response gene to acute γ-irradiation in cell lines [40]. In our study it was not induced significantly above background by acute dose but persistently down-regulated by LDR. Other genes shown here, *RBM3* (RNA binding motif protein 3) and *GRM2* (Glutamate receptor, metabotropic 2) and *DUSP3* (Dual specificity phosphatase 3) have not been previously shown to be affected by radiation in blood and may represent candidate genes for further studies on dose-rate effects in radiation biodosimetry.

A comparison of gene expression changes by dose rate between LDR and acute only, revealed the lowered expression of genes encoding glycolytic enzymes, by the protraction of exposure at 24 h. These genes are involved in glucose metabolism and energy production in cells and the continuous delivery of radiation at LDR may have a dampening effect on the activity of these metabolic functions. In another study using the same LDR, 3.1 mGy/min for 24 h in mice, measurement of metabolites in urine at 48 h after the beginning of exposure showed that citrate in the TCA cycle was decreased by both LDR and acute exposure [41]. In the same study, hexanoylcarnitine and tiglylcarnitine from fatty-acid oxidation pathways were decreased by LDR exposure compared to controls or acute exposure to the same dose, consistent with the perturbations we found reflected in the gene expression data.

The changes observed in blood gene expression after 24 h in the current study support the development of dosimetry signatures to distinguish between dose-rates of exposure, as well as between doses. We performed class predictions by irradiation status and dose-rate (Tables 3 and 4) and dose (Table 5). The classifier gene set that performed best and distinguished between LDR and acute exposure included the LDR-only response genes *DUSP3* and *ID1* (Fig. 3d). This suggests that it may be possible to develop a gene-based signature that can detect protracted exposures without the need for a pre-exposure sample. Further independent validation studies will of course be needed, but such a test could also be used in conjunction with other strictly dosimetric assays, including gene expression or cytogenetic endpoints, to provide a better and more practical biodosimetry assay.

## Conclusions

This study investigated the effects of dose-rate on human blood cell gene expression, over a 24-hour period. Although there were many similarities in immune function and stress response genes, we found that low dose-rate exposure can result in distinctive gene expression patterns compared with acute exposures. Typical p53 gene responses were also seen at all doses delivered by the lower dose rate. We were able to successfully distinguish low dose-rate exposed samples from acute dose exposures, using classification algorithms on our gene expression data. These genes are candidates for further validation studies to develop a gene-based signature that can detect low dose-rate exposures for large-scale biodosimetry.

### Availability of supporting data

The data set supporting the results of this article is available in the NCBI GEO repository [http://www.ncbi.nlm.nih.gov/geo/query/acc.cgi?acc=GSE65292].

## Additional files

Additional file 1: **Custom low density array design for qRT-PCR.**

Additional file 2: **Differential gene expression results, details of Table** 1 **.**

Additional file 3: **Venn diagrams, intersections of genes summarized in Table** 1 **.**

Additional file 4: **qRT-PCR validation results, details of results presented in Figure** 2 **.**

Additional file 5: **Class prediction gene sets, for result in Tables** 3 **,** 4 **and** 5 **.**

## Abbreviations

LDR: Low dose-rate
IND: Improvised nuclear device
RDD: Radioactive dispersal device
