# Supplementary material for: Radiation dose-rate effects on gene expression for human biodosimetry
Source: BMC Med Genomics. 2015 May 12;8:22. doi: 10.1186/s12920-015-0097-x (PMC4472181; doi:10.1186/s12920-015-0097-x)
Supplement: Supplementary file 3 — Venn diagrams, intersections of genes summarized in Table 1 . [file 12920_2015_97_MOESM3_ESM.pdf]

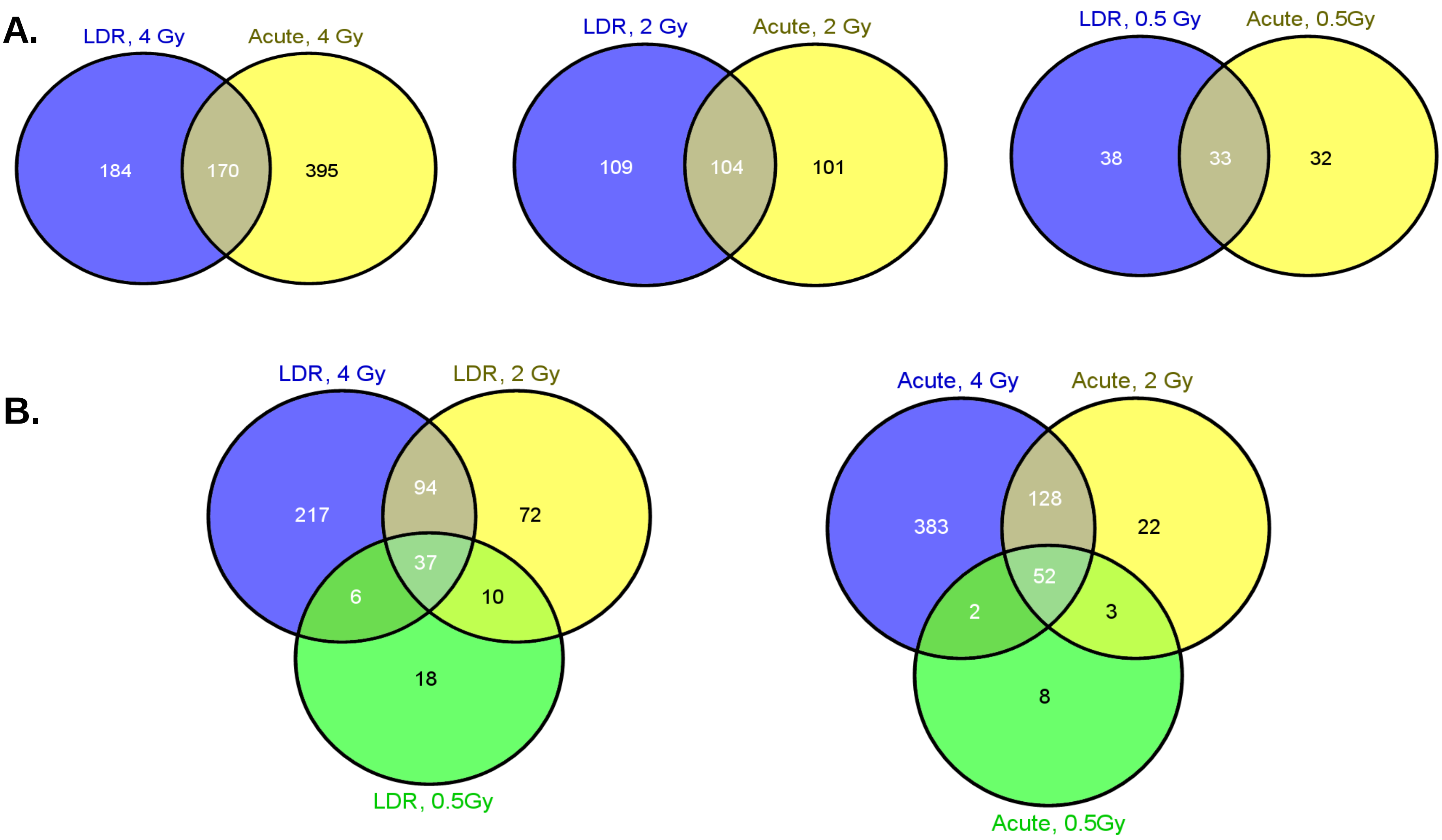

Additional file 3 Venn diagrams showing the intersect between class comparisons, summarized in Table 1 (detailed gene lists in Additional file 2). **A.** intersection between LDR and acute at 4.45 Gy, 2.23 Gy and 0.56 Gy. **B.** Intersection between gene sets responding to different doses, LDR on the left, Acute on the right.
Venn diagrams were created using the tool available at Venny (*http://bioinfogp.cnb.csic.es/tools/venny/index.html.)*
